# Supplementary material for: Topological ultranodal pair states in iron-based superconductors
Source: Nat Commun. 2020 Jan 27;11:523. doi: 10.1038/s41467-020-14357-2 (PMC6985224; doi:10.1038/s41467-020-14357-2)
Supplement: Supplementary file 1 — Supplementary Information [file 41467_2020_14357_MOESM1_ESM.pdf]

Supplementary Material: Topological Ultranodal pair states in iron-based superconductors

Setty et al

In this Supplementary Material, we provide numerical results for the two hole-pocket model and other additional details to support the main claims in our paper.

Supplementary Note 1. 2-pocket model

We begin by discussing results for the two hole-pocket model (centered around Γ) with a pairing ansatz of the form $\Delta_j(\mathbf{k}) = \Delta_{ja}(\mathbf{k}) + \Delta_j$, where $\Delta_{ja}(\mathbf{k})$ (Δ_j) is the anisotropic (isotropic) component on hole pocket $j = 1, 2$. We vary the isotropic components on the second hole pocket for a fixed inter-pocket pairing Δ_0. Supplementary Fig. 1 shows a plot of C_V (a) and C_V/T (b) as a function of temperature for different values of the singlet isotropic component of the gap on the second band Δ_2. We have chosen the TRSB component (δ) for each curve to be non-zero and equal to the inter-band pairing order parameter which is set to $\Delta_0 = 0.3$. The anisotropic, singlet pairing component on both the bands (Δ_{1a}, Δ_{2a}) is

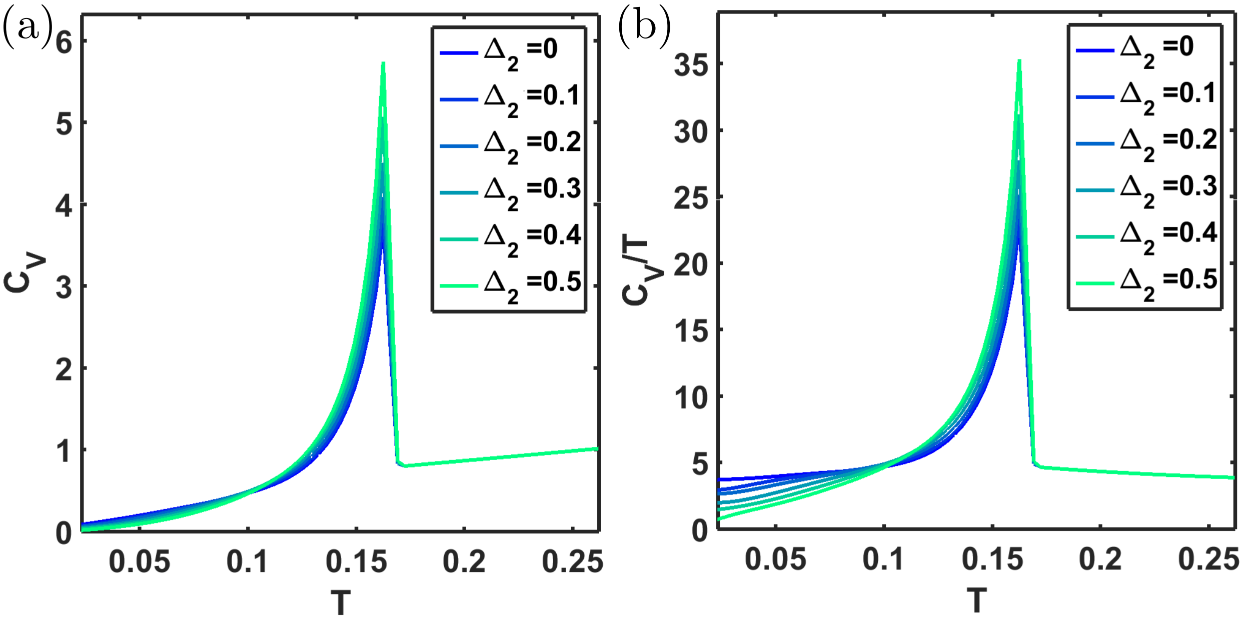

Supplementary Figure 1. Temperature dependence of the specific heat C_V (left) and $\frac{C_V}{T}$ (right) as a function of the isotropic gap parameter on the second band Δ_2. The inter-band gap parameter is chosen to be $\Delta_0 = 0.3$ and the time-reversal broken component $\delta = \Delta_0$ for each panel. The singlet anisotropic gap components on the first (second) band is fixed at $\Delta_{1a} = 0.1$ ($\Delta_{2a} = 0.3$), and the isotropic gap on the first band $\Delta_1 = 0.6$.

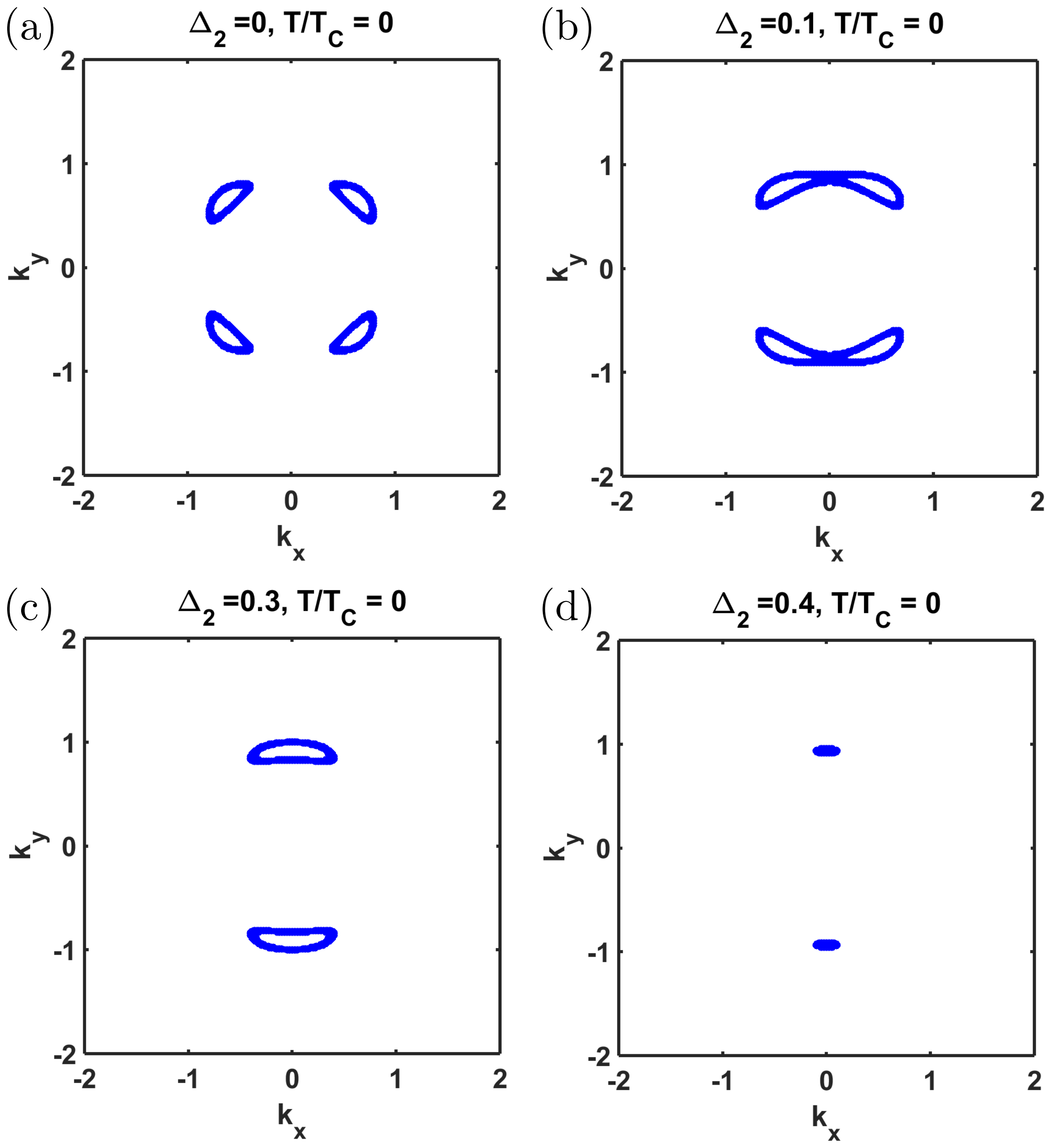

Supplementary Figure 2. Fermi surfaces for different values of the isotropic gap on the second band, Δ_2, at zero temperature. Above $\Delta_2 = 0.4$, the Fermi surface is fully gapped. The inter-band gap parameter is chosen to be $\Delta_0 = 0.3$ and the time-reversal broken component $\delta = \Delta_0$ for each panel. The singlet anisotropic gap components on the first (second) band is fixed at $\Delta_{1a} = 0.1$ ($\Delta_{2a} = 0.3$), and the isotropic gap on the first band $\Delta_1 = 0.6$. Note the C_2 symmetry of the nodes, consistent with ARPES.

Inter-orbital	TRSB	Spin triplet	Z_2
N	N	N	0
N	Y	N	0
Y	N	N	0
Y	Y	N	0
N	Y	Y	0
Y	Y	Y	1, if $\Delta_0 > \Delta_i(\mathbf{k})$
N	N	Y	Undefined
Y	N	Y	0

Supplementary Table I. Supplementary Table summarizing the various possible gap combinations and their Z_2 invariants.

chosen to be 0.1 and 0.3 respectively. All the C_V curves show a superconducting jump at T_c and go to zero at zero temperature. However, the exponent of the temperature dependence varies drastically with Δ_2. This is shown in Supplementary Fig. 1 (b) where C_V/T saturates at zero temperatures for $\Delta_2 \leq 0.4 \equiv \Delta_{2c}$ and goes to zero above Δ_{2c}. This low temperature behavior can be understood from the corresponding zero energy contours shown in Supplementary Fig. 2 for T close to zero and different Δ_2. Above the critical value of the isotropic gap Δ_{2c}, the FS is fully gapped, while a Bogoliubov surface exists in two dimensions for Δ_2 equal to or below Δ_{2c}. Close to Δ_{2c}, the Bogoliubov surface shrinks continuously to a point. The low-energy Bogoliubov excitations for $\Delta_2 \leq \Delta_{2c}$ give rise to the residual specific heat at low temperatures, and the thermodynamic properties of the system resemble that of a normal metal.

Supplementary Note 2. Summary table

Supplementary Table I summarizes the various possible gap combinations and their respective Z_2 invariants. "Y" ("N") represents whether the corresponding pairing combination is present (absent) in the model Hamiltonian. In each case, one can determine the Pfaffian of

the CP symmetric Hamiltonian and determine its Z_2 invariant depending on whether there is a sign change. The ultranodal superconductor with Bogoliubov surfaces exists only in the presence of a non-zero interorbital pairing along with TRSB and higher spin-angular momentum pairing (e.g. triplet). As mentioned in the main text, there are two equivalent physical systems which yields the same Pfaffian and hence low-energy excitation spectrum. While the first pairing Hamiltonian is described in the main text, the second contains an interband, TRSB spin-triplet-orbital-singlet, and a TRS spin-singlet-orbital-triplet component. In second quantized notation, it takes the form

$$\begin{aligned}\hat{H}_\Delta = \delta \sum_{\mathbf{k},i\neq j} \left(c^\dagger_{\mathbf{k}i\uparrow} c^\dagger_{-\mathbf{k}j\downarrow} + c^\dagger_{\mathbf{k}i\downarrow} c^\dagger_{-\mathbf{k}j\uparrow} \right) + h.c. - (i \leftrightarrow j) \\ + \Delta_0 \sum_{\mathbf{k},i\neq j} \left(c^\dagger_{\mathbf{k}i\uparrow} c^\dagger_{-\mathbf{k}j\downarrow} - c^\dagger_{\mathbf{k}i\downarrow} c^\dagger_{-\mathbf{k}j\uparrow} \right) + h.c. + (i \leftrightarrow j) \\ + \sum_{\mathbf{k},i} \Delta_i(\mathbf{k}) \left(c^\dagger_{\mathbf{k}i\uparrow} c^\dagger_{-\mathbf{k}i\downarrow} - c^\dagger_{\mathbf{k}i\downarrow} c^\dagger_{-\mathbf{k}i\uparrow} \right) + h.c. \end{aligned} \tag{1}$$

It is easy to check that the above pairing Hamiltonian, along with the kinetic part $\hat{H}_0$, has the same Pfaffian presented in the main text for the two-pocket model.

Supplementary Note 3. Conventional d-wave order parameter

The real system $FeSe_{1-x}S_x$ discussed in the main text exhibits quite small Fermi surfaces at the Γ point as expected from its smooth connection to the nematic FeSe material. Given that the s and d wave instabilities are found to be competing in Fe-based systems in general when calculated using a spin-fluctuation driven mechanism, it is possible that the systems undergoes a transition towards a d wave state as a function of sulfur doping. There are several reasons to believe that this could happen in principle. First, upon lowering of the nematic order, the coupling between the s-wave instability and the d-wave instability decreases until it eventually vanishes at the nematic phase transition at $x \sim 0.23$. In consequence, the relative competition of the leading and subleading superconducting instability increases. Additionally, the $FeSe_{1-x}S_x$ system tends to be less correlated. Considering that the d_{xy} orbital as the orbital with the strongest correlations should achieve significant coherence upon reducing correlations, it is expected that (π, π) fluctuations are getting relatively

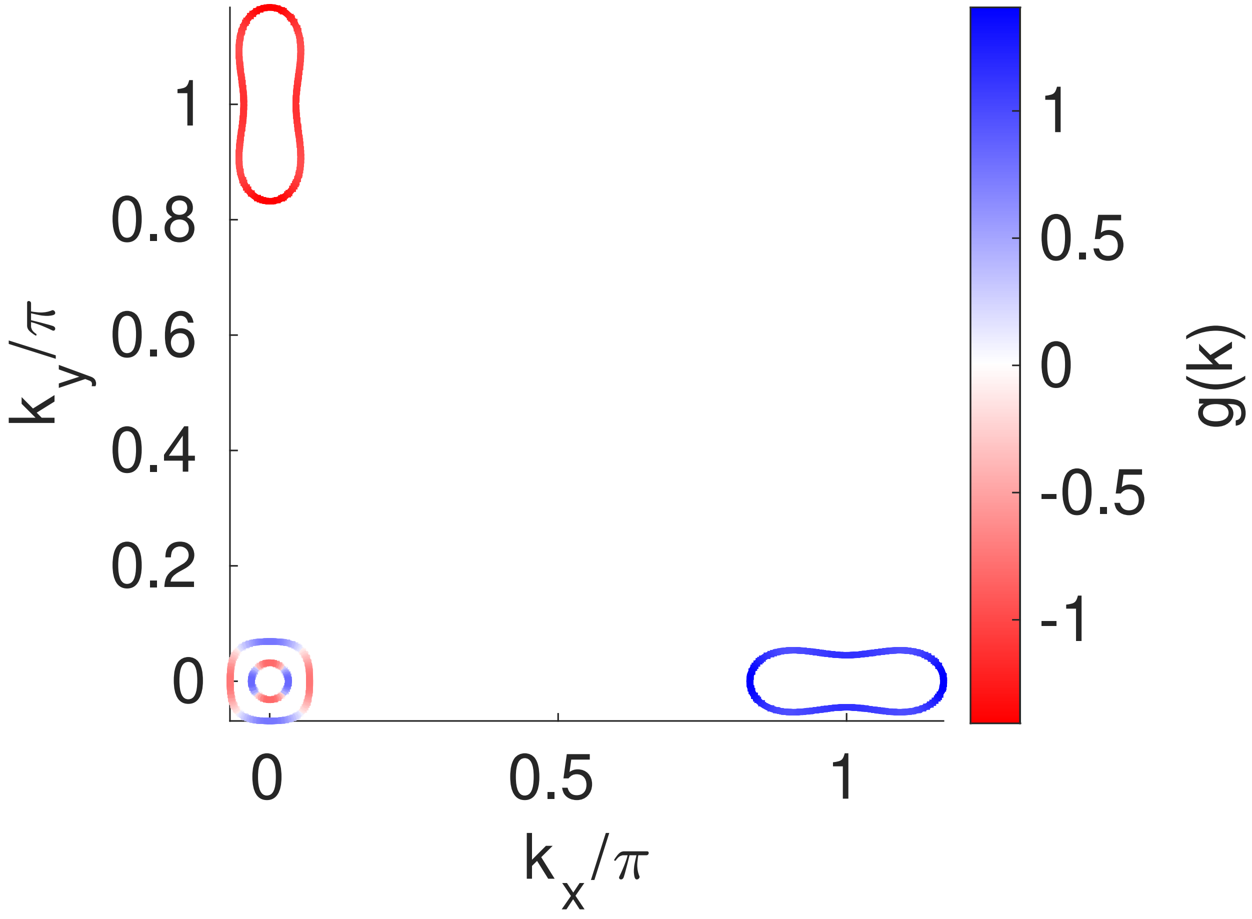

Supplementary Figure 3. Superconducting order parameter on the Fermi surface as obtained from modified spin-fluctuation pairing calculations[1] for a model of $FeSe_{1-x}S_x$, $U = 0.54$, $J = U/6$.

enhanced and the corresponding d-wave channel can become the leading superconducting instability. In fact, it is possible to achieve such a situation from a microscopic calculation using a spin-fluctuation pairing calculation with reasonable choices of the quasiparticle weights according the trends just outlined. For this, we have adopted a two dimensional version of electronic structure for FeSe from Ref. 2 by ignoring the hoppings in z direction and removing the orbital order term as expected beyond the nematic transition in the $FeSe_{1-x}S_x$ system. Following the trends imposed by reduced correlations[3], we choose as quasiparticle weights $\sqrt{Z_l} = [0.69804, 0.98827, 0.83894, 0.83894, 0.83072]$ and find a leading d-wave instability as shown in Supplementary Fig. 3. Indeed, the Γ centered Fermi surfaces exhibit a small k_F and thus the d-wave order parameter with a nodal point at $\mathbf{k} = 0$ is small in magnitude on these Fermi surface parts, providing a significant density of low energy

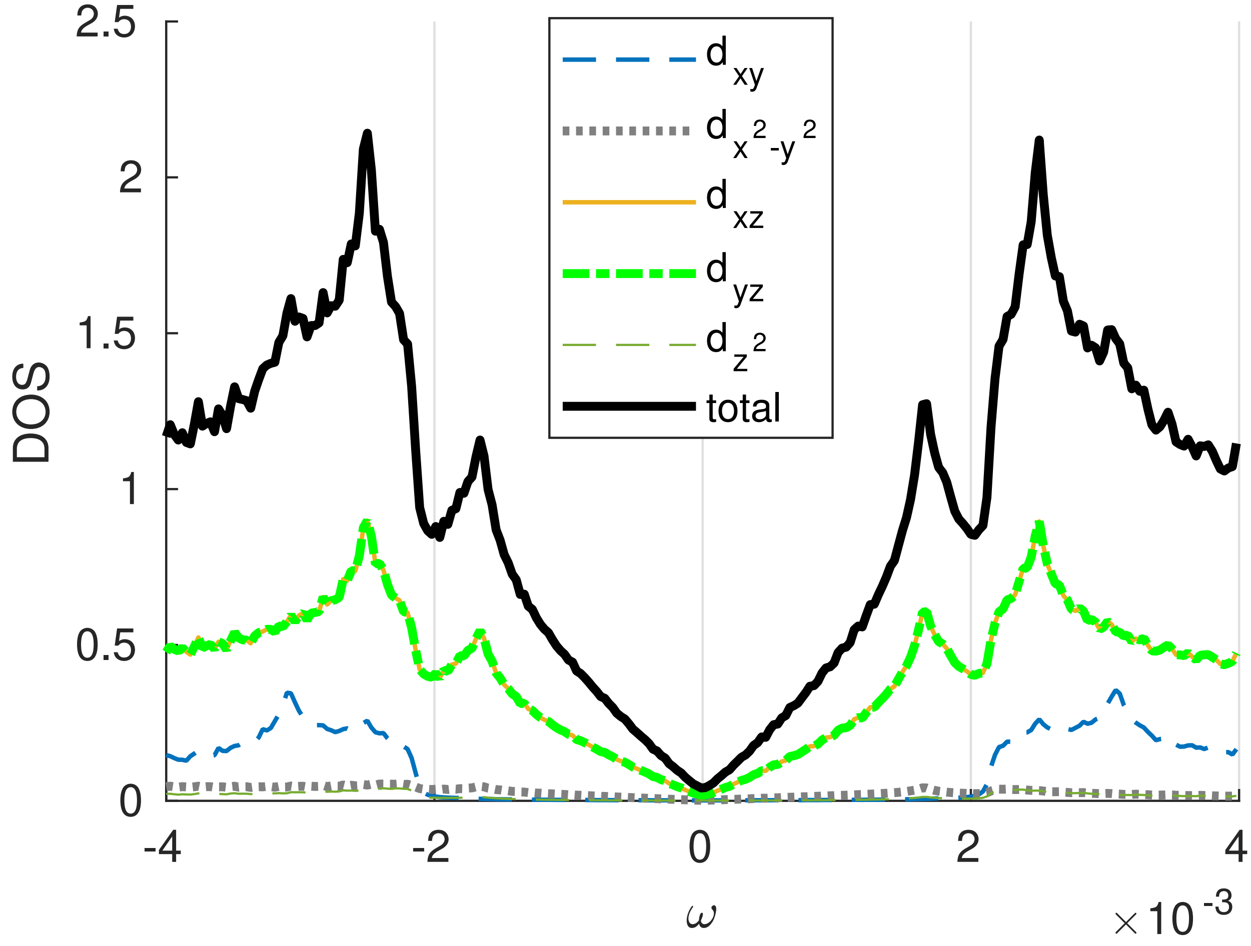

Supplementary Figure 4. Density of states as calculated from the d-wave superconducting order parameter in the model for $FeSe_{1-x}S_x$ showing low energy excitations in the d_{xz} and d_{yz} orbital components that give rise to the states at the Fermi surface around the Γ point.

excitations in the superconducting state. This situation is very similar to the one discussed in the main text (parameter set A), and yields a V-shaped density of states at low energies, see Supplementary Fig. 4. A corresponding calculation of the entropy as a function of temperature by imposing a superconducting order parameter $\Delta(\mathbf{k}, T) = \tilde{\Delta}(T)g(\mathbf{k})$, where $g(\mathbf{k})$ is the gap symmetry function as shown in Supplementary Fig. 3 and $\tilde{\Delta}(T)$ simply follows the temperature dependence of a mean field superconducting order parameter, yields significant contributions to the specific heat at low temperatures, see Supplementary Fig. 5. However, the quasiparticle dispersion has only nodal points (in two dimensions) and therefore yields a linear behavior of C_V/T vs. T, and can never achieve a finite value for $T \to 0$ in the clean case, unlike the situation of a Bogoliubov Fermi surface as discussed in

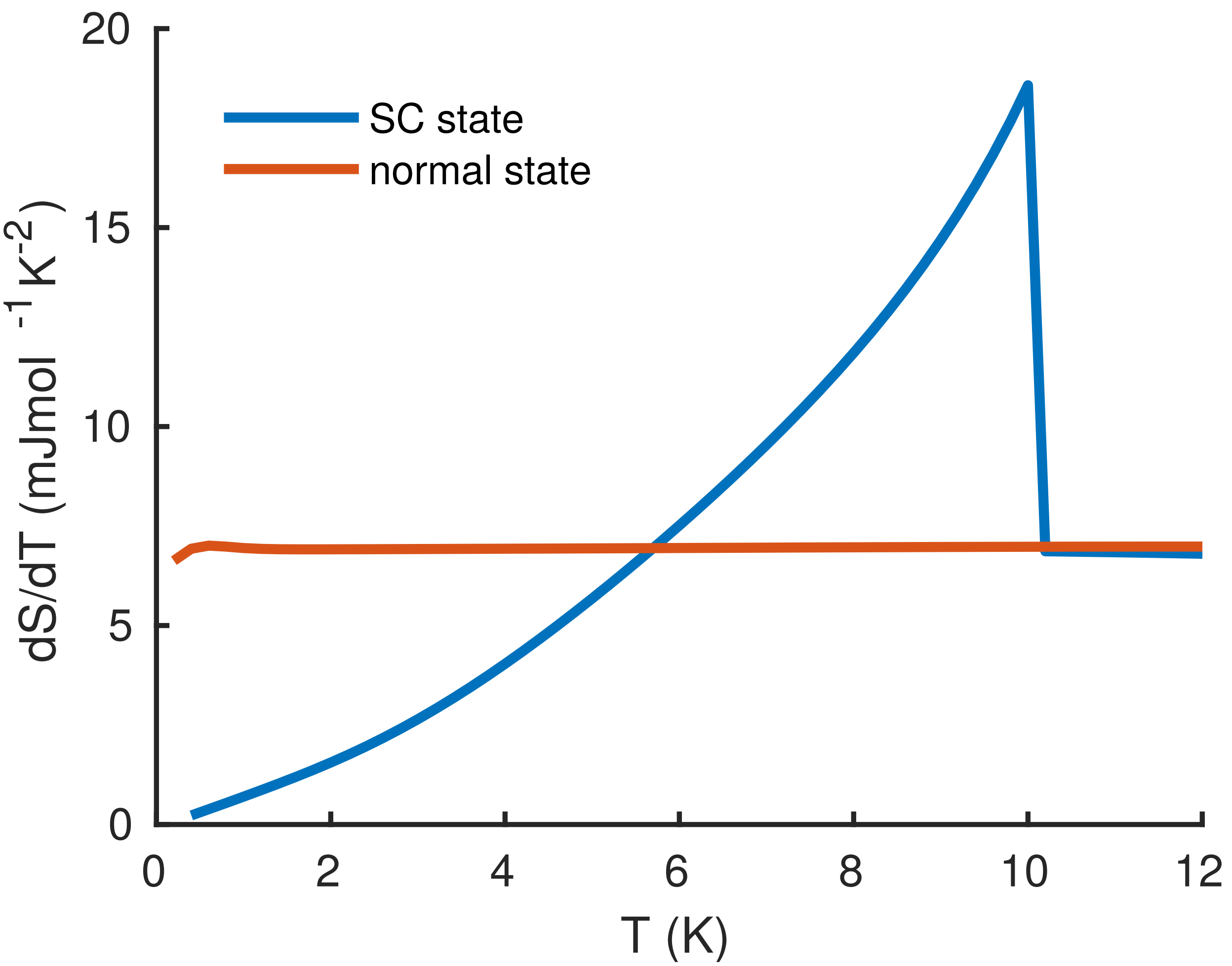

Supplementary Figure 5. Specific heat C_V/T for the d-wave superconducting state exhibiting a linear dependence at low temperatures, but no finite value at $T \to 0$. The superconducting state curve does not appear to reflect the excess quasiparticle density on the small hole pockets that was anticipated by the authors of Ref. 4.

the main text. In summary, such a proposed d-wave state with small Fermi surface pockets in a realistic model for the band structure cannot account for the experimental observations of a finite C_V/T.

Supplementary Note 4. Symmetry operators for n bands

For an $n-$band model, with respect to the basis chosen in the main text where the orbitals/bands transform trivially under time reversal, the corresponding unitary matrices for

CPT symmetries take the form $U_P = \pi_0 \otimes \mathbb{I}_n \otimes \sigma_0$, $U_T = \pi_0 \otimes \mathbb{I}_n \otimes i\sigma_y$ and $U_C = \pi_x \otimes \mathbb{I}_n \otimes \sigma_0$. Here $\mathbb{I}_n$ is an $n \times n$ unit matrix.

We now provide explicit forms of the matrix operators appearing in the main text for the two pocket case. $\hat{0}_n$ means a $n \times n$ matrix of zeroes.

$$H(\mathbf{k}) = \begin{bmatrix} \epsilon_1(\mathbf{k})\mathbb{I}_2 & \hat{0}_2 & i\Delta_1(\mathbf{k})\sigma_y & \Delta_0\mathbb{I}_2 + \delta\sigma_z \\ \hat{0}_2 & \epsilon_2(\mathbf{k})\mathbb{I}_2 & -\Delta_0\mathbb{I}_2 - \delta\sigma_z & i\Delta_2(\mathbf{k})\sigma_y \\ -i\Delta_1(\mathbf{k})\sigma_y & -\Delta_0\mathbb{I}_2 - \delta\sigma_z & -\epsilon_1(\mathbf{k})\mathbb{I}_2 & \hat{0}_2 \\ \Delta_0\mathbb{I}_2 + \delta\sigma_z & -i\Delta_2(\mathbf{k})\sigma_y & \hat{0}_2 & -\epsilon_2(\mathbf{k})\mathbb{I}_2 \end{bmatrix} \tag{2}$$

$$C = \begin{bmatrix} \hat{0}_4 & \mathbb{I}_4 \\ \mathbb{I}_4 & \hat{0}_4 \end{bmatrix} \tag{3}$$

$$T = \begin{bmatrix} i\sigma_y & \hat{0}_2 & \hat{0}_2 & \hat{0}_2 \\ \hat{0}_2 & i\sigma_y & \hat{0}_2 & \hat{0}_2 \\ \hat{0}_2 & \hat{0}_2 & i\sigma_y & \hat{0}_2 \\ \hat{0}_2 & \hat{0}_2 & \hat{0}_2 & i\sigma_y \end{bmatrix} \tag{4}$$

Finally the parity operator is simply an identity matrix $P = \mathbb{I}_8$.

Supplementary Note 5. Stability of the Bogoliubov Fermi Surface

As stated in the main text, theories with pure interpocket pairs are thought to be generally unstable as they can exhibit negative superfluid density[5]. In this section, we present a calculation to confirm that the presence of intrapocket pairs stabilizes the system. More specifically, we show a situation where stable Bogoliubov Fermi surfaces exists. In other words, we demonstrate that the quasiparticle spectrum can remain gapless in the topological phase while the superfluid density is positive. We only present a short summary of our conclusions; the broader question in which circumstances a negative superfluid density occurs, is interesting on its own, and will be addressed in more detail in future work.

We begin with the paramagnetic current operator for a two band system

$$\mathbf{j}^P(\mathbf{q}) = -e \sum_{\mathbf{k},i,s} \frac{1}{m_i}(\mathbf{k} + \frac{\mathbf{q}}{\mathbf{2}}) c^\dagger_{\mathbf{k}is} c_{\mathbf{k}+\mathbf{q}is} \tag{5}$$

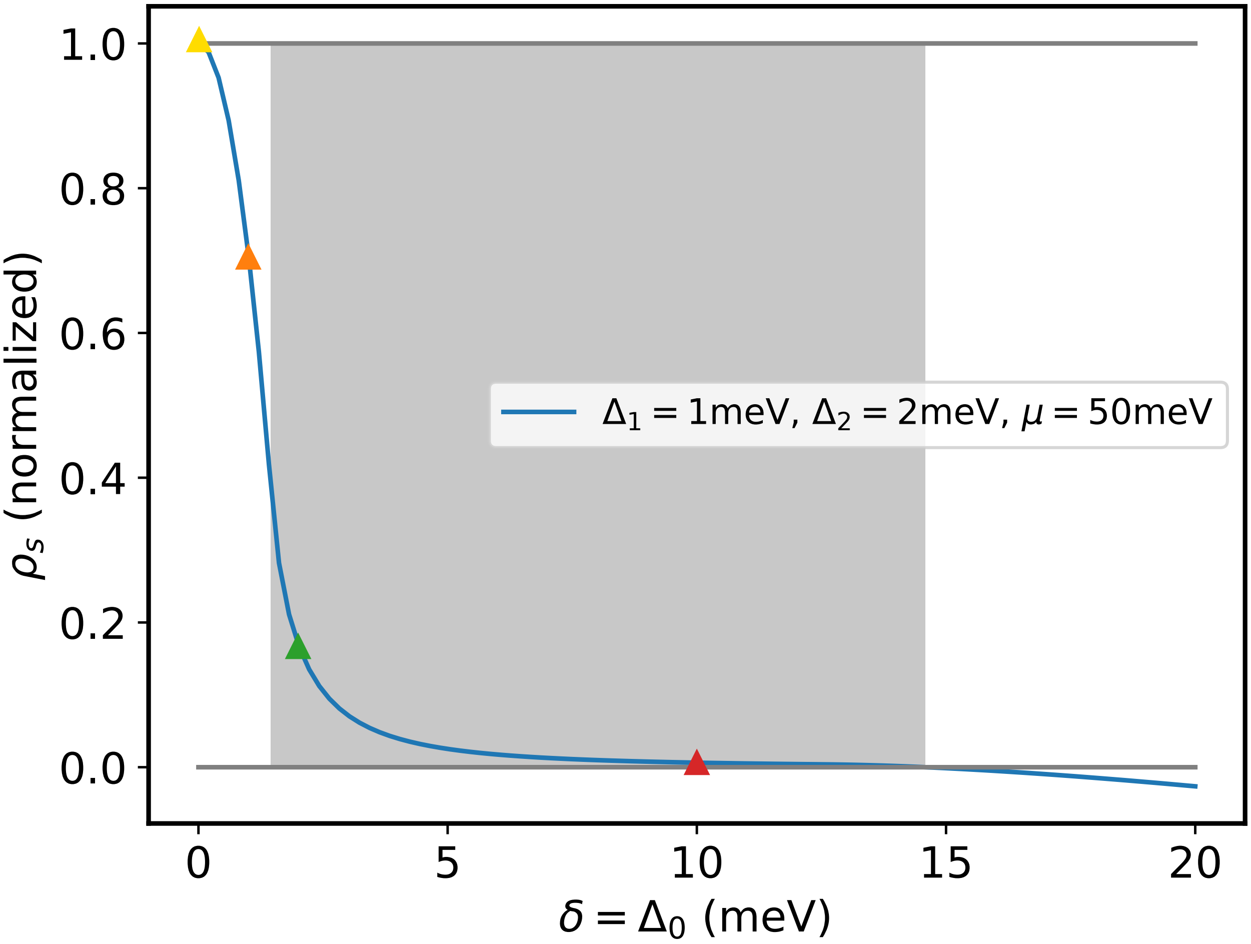

Supplementary Figure 6. Superfluid density as a function of $\delta = \Delta_0$ when $m_1 = -m_2$. The triangles mark the values of δ for which the quasiparticle dispersions are plotted in Supplementary Fig. 7. A Bogoliubov Fermi Surface exists with a positive superfluid density for in the range of interband pairings that is shaded gray, including $\delta = 2$meV (green triangle) and $\delta = 10$meV (red triangle).

which can be written as

$$\mathbf{j}^P(\mathbf{q}) = -\frac{e}{2}\sum_{\mathbf{k}}(\mathbf{k} + \frac{\mathbf{q}}{\mathbf{2}})\psi^\dagger_{\mathbf{k}}\begin{pmatrix} \frac{1}{m_1} & \\ & \frac{1}{m_2} \end{pmatrix}\psi_{\mathbf{k+q}}, \tag{6}$$

$$= -\frac{e}{2}\sum_{\mathbf{k}}(\mathbf{k} + \frac{\mathbf{q}}{\mathbf{2}})\psi^\dagger_{\mathbf{k}} M \psi_{\mathbf{k+q}}, \tag{7}$$

where $\psi^\dagger_{\mathbf{k}} = \left(c^\dagger_{\mathbf{k},1,s}, c^\dagger_{\mathbf{k},2,s}\right)$ is the vector electron annihilation operator and we introduced the matrix of inverse masses $M = \text{diag}(1/m_1, 1/m_2)$ The time-ordered current-current correla-

tion function is then

$$-i\langle\mathcal{T}j_i^P(\mathbf{q},\tau)j_j^P(-\mathbf{q},0)\rangle = i\left(\frac{e}{2}\right)^2\sum_{\mathbf{k},\mathbf{k}'}(\mathbf{k}+\frac{\mathbf{q}}{\mathbf{2}})_i(\mathbf{k}'+\frac{\mathbf{q}}{\mathbf{2}})_j\mathrm{Tr}[MG(\mathbf{k}+\mathbf{q},\tau)MG(\mathbf{k},-\tau)]\delta_{\mathbf{k},\mathbf{k}'}\,, \tag{8}$$

where $G(\mathbf{k},\tau)$ is the quasiparticle Green function. With this definition, the current response kernel is defined by

$$J_i(\mathbf{q},\omega) = -\frac{c}{4\pi}K_{ij}(\mathbf{q},\omega)A_j(\mathbf{q},\omega), \tag{9}$$

$$K = K^P + K^{\mathrm{dia}}, \tag{10}$$

where $A_i(\mathbf{q},\omega)$ is the vector potential, K^P and K^{dia} are the paramagnetic and diamagnetic

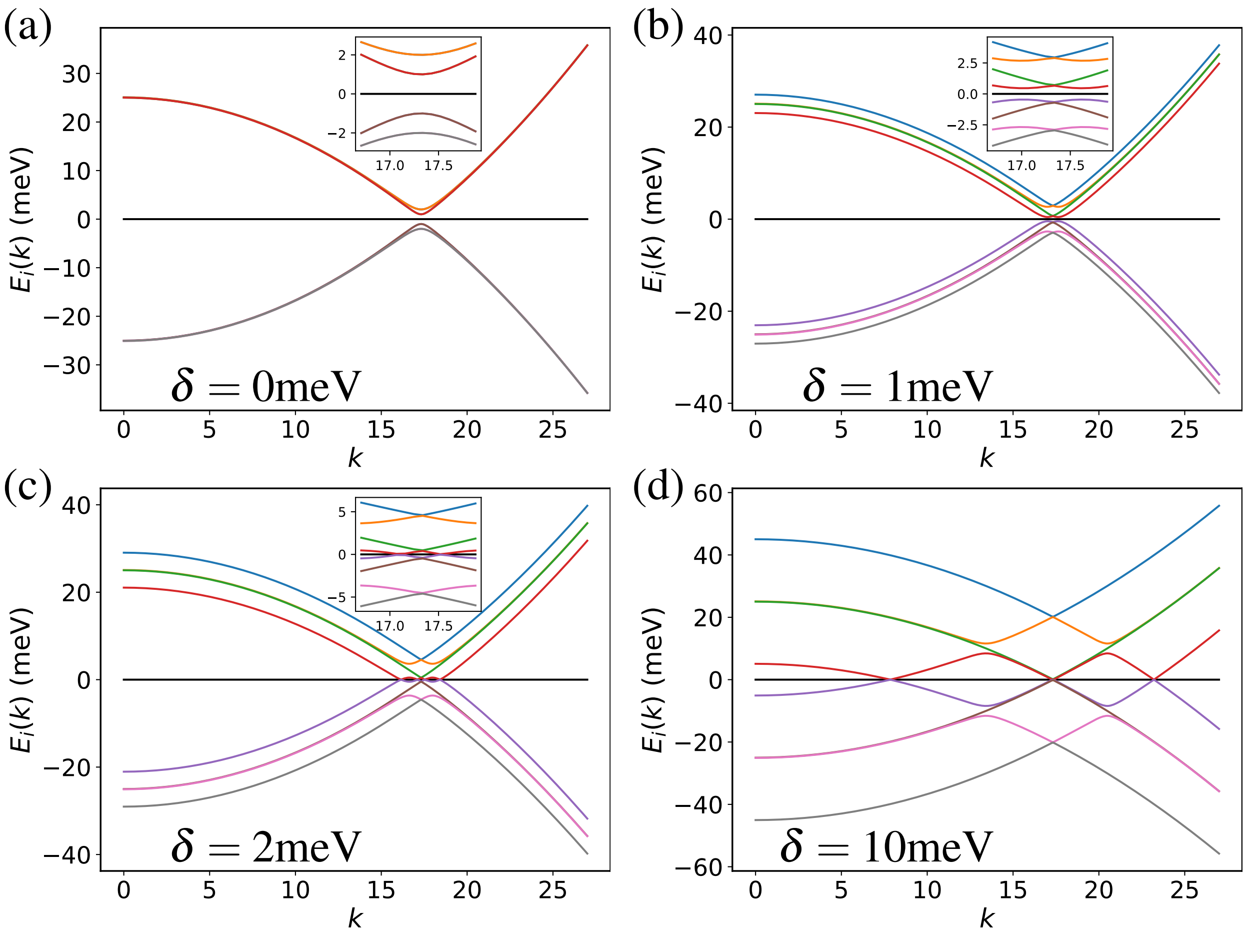

Supplementary Figure 7. Plots of the quasiparticle band energies $E_i(k)$ for different values of $\Delta_0 = \delta$. (a) $\delta = 0$, (b) $\delta = 1$meV, (c) $\delta = 2$ meV, (d) $\delta = 10$meV. A Bogoliubov Fermi Surface exists with a positive superfluid density for $\delta = 2$meV and $\delta = 10$meV.

responses, respectively. The diamagnetic kernel is given as usual for parabolic bands as

$$K^{\rm dia} = \frac{4\pi e^2}{c^2} \sum_{\alpha} \frac{n_\alpha}{|m_\alpha|}, \tag{11}$$

where n_α is the density of electrons (holes) in electronlike (holelike) band α in the normal state, and the paramagnetic kernel is

$$K^P_{ij}(\mathbf{q} \to 0, \omega = 0) = \frac{\pi e^2}{c^2} \sum_{\mathbf{k}} k_i k_j \frac{1}{\beta} \sum_{\nu_n} {\rm Tr}[MG(\mathbf{k}, i\nu_n) MG(\mathbf{k}, i\nu_n)]. \tag{12}$$

The normalized superfluid density ρ_s is then defined as $1 + \frac{K^P(\mathbf{q}\to 0, \omega=0, \delta=\Delta_0)}{K^{\rm dia}(\delta=\Delta_0=0)}$. Here it can be shown that for $\delta \neq 0$, the diamagnetic response is approximately a constant provided the interband pairing is small compared to the Fermi energy. In the limit of $\delta, \Delta_0 \to 0$, the superfluid density reduces to that of a conventional superconductor. A plot of ρ_s as a function of interband pairing $\Delta_0 = \delta$ is shown in Supplementary Fig. 6 for the case of two band masses with opposite sign $m_2 = -m_1$. The triangles on the curve mark values of the inter-band pairing for which the quasiparticle dispersions are shown to observe the presence of Bogoliubov Fermi surfaces. These dispersions are plotted in the panels of Supplementary Fig. 7. For the yellow triangle, there is no Bogoliubov surface as the quasiparticle spectrum is fully gapped. However, for a range of inter-band pairings including the green ($\delta = 2$meV) and red ($\delta = 10$meV) triangles, the superfluid density is positive and at the same time a Bogoliubov surface exists. Hence this shows that there is a region of parameter space where, for a non-zero value of the intra-pocket gap, the Bogoliubov surface is stable. For simple harmonic gap functions, the region of stability of the topological phase is enhanced by the creation of strong anisotropy in the intraband gap function, as considered in the main text.

Supplementary References

[1] Andreas Kreisel, Brian M. Andersen, P. O. Sprau, A. Kostin, J. C. Séamus Davis, and P. J. Hirschfeld, "Orbital selective pairing and gap structures of iron-based superconductors," Phys. Rev. B **95**, 174504 (2017).

[2] P. O. Sprau, A. Kostin, A. Kreisel, A. E. Böhmer, V. Taufour, P. C. Canfield, S. Mukherjee,

P. J. Hirschfeld, B. M. Andersen, and J. C. S. Davis, "Discovery of orbital-selective Cooper pairing in FeSe," Science **357**, 75–80 (2017), arXiv:1611.02134 [cond-mat.supr-con].

[3] P. Reiss, M. D. Watson, T. K. Kim, A. A. Haghighirad, D. N. Woodruff, M. Bruma, S. J. Clarke, and A. I. Coldea, "Suppression of electronic correlations by chemical pressure from FeSe to FeS," Phys. Rev. B **96**, 121103 (2017).

[4] Yuki Sato, Shigeru Kasahara, Tomoya Taniguchi, Xiangzhuo Xing, Yuichi Kasahara, Yoshifumi Tokiwa, Youichi Yamakawa, Hiroshi Kontani, Takasada Shibauchi, and Yuji Matsuda, "Abrupt change of the superconducting gap structure at the nematic critical point in $FeSe_{1-x}S_x$," Proceedings of the National Academy of Sciences **115**, 1227–1231 (2018).

[5] Wan, Yuan and Wang, Qiang-Hua, "Pairing symmetry and properties of iron-based high-temperature superconductors," EPL **85**, 57007 (2009).
